# Enhanced photoelectrochemical properties of nanocrystalline TiO_2_ electrode by surface sensitization with Cu_x_O quantum dots

**DOI:** 10.1038/s41598-017-05645-x

**Published:** 2017-07-13

**Authors:** Jiajia Tao, Zhaoqi Sun, Yunlang Cheng, Miao Zhang, Jianguo Lv, Shiwei Shi, Gang He, Xishun Jiang, Xiaoshuang Chen, Xingzhi Wang, Zhuang Wang, Zezhou Gong

**Affiliations:** 1 School of Physics & Materials Science, Anhui University, Hefei, 230601 P.R. China; 2 School of Electronic & Information Engineering, Hefei Normal University, Hefei, 230601 P.R. China; 3 School of Mechanical & Electronic Engineering, Chuzhou University, Chuzhou, 239000 P.R. China; 4 National Laboratory for Infrared Physics, Shanghai Institute of Technical Physics, Chinese Academy of Sciences, Shanghai, 200083 P.R. China

## Abstract

Nanoporous anatase TiO_2_ films were fabricated by a screen-printing method, and Cu_x_O quantum dots (QDs) were deposited on the TiO_2_ films through successive ionic layer adsorption and reaction (SILAR). The amount of Cu_x_O QDs on the TiO_2_ films are controlled by changing the number of SILAR cycles. The morphology, microstructure, optical, and photoelectrochemical properties of different Cu_x_O sensitized TiO_2_ films (Cu_x_O/TiO_2_) were investigated in detail. The nanoporous TiO_2_ film offers a large surface area for anchoring QDs. QD deposited samples exhibited a significant improvement in photoelectrochemical performance than the bare of TiO_2_. Cu_x_O/TiO_2_, prepared with 7 SILAR cycles, showed the best photoelectrochemical properties, where the photocurrent density was enhanced to 500.01 μA/cm^2^ compared with 168.88 μA/cm^2^ of bare TiO_2_ under visible light. These results indicate that the designed Cu_x_O/TiO_2_ structure possesses superior charge separation efficiency and photoelectrochemical properties.

## Introduction

Titanium dioxide (TiO_2_) has attracted great interest for water splitting^1, 2^, quantum dot-sensitized solar cells^3, 4^, optical sensors^5, 6^, photocatalytic degradation^7^ and other applications^8–10^, due to its unique optical and photoelectric properties. Nevertheless, the intrinsic band gap of TiO_2_ (3.0 eV for rutile and 3.2 eV for anatase) limits its photoelectrochemical utility^11^. Specifically, the wide band gap restricts the photoresponse of TiO_2_ to only ultraviolet region, with the wavelengths below 380 nm, which constitutes less than 5% of the solar spectrum. Therefore, many studies have been conducted to extend the TiO_2_ optical absorption range to the visible light region, in order to expand the portion of the solar spectrum for which TiO_2_ can be utilized.

In recent years, quantum dots (QDs) have become increasingly attractive for similar applications as TiO_2_, because of their unique electronic and optical properties^12, 13^, such as their high extinction coefficients^14, 15^, tunable band gap^16, 17^, multiple exciton generation^18, 19^, and an expandable optical absorption range by controlling particle size^20, 21^. Various QDs, including CdS^22^, CdSe^23^, PbS^24^, PbSe^25^, and so on^26, 27^, have been thoroughly studied. Although these studies have demonstrated that QDs-sensitized TiO_2_ can efficiently absorb visible light, inherent disadvantages of these QDs, such as a fixed visible light absorption range, the toxicity of elements, and the use of rare elements, limit their applications in biology, environment, medicine, and solar cells^28^. As a result, it is important to discover environmentally friendly QD materials. Recently, the development of copper oxides as both the core photocatalytic material and the photoelectrochemical material has drawn increasing amounts of attention^29–31^. Copper materials, which are multifunctional p-type semiconductors, with a band gap ranging between 1.5 and 2.4 eV^32^, are environmentally friendly and can absorb in the visible light region^33, 34^. Until now, to the best of our knowledge, previous studies have not reported the surface sensitization of a nanocrystalline TiO_2_ electrode with Cu_x_O QDs.

Unlike many syntheses of QDs, such as electrode deposition^35^, photocatalytic reduction^36^, and sol-gel method^37^, our study uses a novel technique to fabricate Cu_x_O QDs via successive ionic layer adsorption and reaction (SILAR) method^38^. SILAR is a simple fabrication methodology that combines successive layer adsorption with chemical redox reaction. The typical process involves successively immersing the TiO_2_ materials in ${\text{Cu}(\text{NH}}_{3}{)}_{4}^{2+}$ and H_2_O_2_ aqueous solutions successively for as many cycles as desired to achieve a uniform deposition of Cu_x_O QDs. Not only is the overall process environmentally friendly, cost-effective, and can be carried out in normal atmospheric pressure and room temperature, but the density of QDs can also be easily controlled by merely varying the number of deposition cycles.

In this study, we report the preparation of nanoporous anatase TiO_2_ films on transparent conductive fluorine-doped tin oxide (FTO) substrates by screen-printing and the subsequent deposition of Cu_x_O QDs on the TiO_2_ films via SILAR. The number of deposition cycles, and thus QD loading density, was varied to investigate the effect on the photoelectrochemical properties of nanocrystalline TiO_2_ electrode and derive the optimal density of Cu_x_O QDs that enhanced the photoelectrochemical activity. The sample was also recycled 7 times to demonstrate its improved absorbance in the visible range and enhanced photoelectrochemical properties.

## Experimental

### Preparation of TiO_2_ films

All the chemical reagents were used as received. The colloidal were prepared by hydrolysis of titanium tetraisopropoxide as described by elsewhere^39^. The TiO_2_ films were synthesized directly on transparent fluorine-doped tin oxide (FTO, TEC-8, LOF) conducting glass substrates by screen-printing, followed by calcining the samples at 500 °C for 30 min in air.

### Immobilization of Cu_x_O quantum dots onto TiO_2_ films

The Cu_x_O QDs were deposited onto the TiO_2_ photoanodes *in situ* by successive SILAR cycles. For Cu_x_O deposition, the TiO_2_ photoanodes were successively immersed in two different solutions for 5 min in each solution, first, in ${\text{Cu}(\text{NH}}_{3}{)}_{4}^{2+}$ and second in H_2_O_2_ aqueous solutions. Following each immersion, the TiO_2_ photoanodes were rinsed with deionized water. The ${\text{Cu}(\text{NH}}_{3}{)}_{4}^{2+}$ were resulting from Cu(AC)_2_ and NH_3_ $\bullet$H_2_O. These sequential steps are considered as one SILAR cycle. The SILAR cycle was repeated for 0, 3, 5, 7, and 9 times. Samples are denoted as S0, S3, S5, S7, and S9, respectively. The relevant reactions for preparing Cu_x_O QDs can be written as follows^40^:

1 $${\rm{Cu}}{({\rm{AC}})}_{2}+6{{\rm{NH}}}_{3}\bullet {{\rm{H}}}_{2}{\rm{O}}\to {\rm{Cu}}{({{\rm{NH}}}_{3})}_{4}^{2+}+2{{\rm{NH}}}_{4}{\rm{AC}}+2{{\rm{OH}}}^{-}+4{{\rm{H}}}_{2}{\rm{O}}$$

2 $${\rm{Cu}}{({{\rm{NH}}}_{3})}_{4}^{2+}+{{\rm{H}}}_{2}{{\rm{O}}}_{2}+2{{\rm{OH}}}^{-}\to {\rm{Cu}}+{{\rm{O}}}_{2}+4{{\rm{NH}}}_{3}+2{{\rm{H}}}_{2}{\rm{O}}$$

### Characterization techniques

Microstructures and the crystallinity of Cu_x_O sensitized TiO_2_ films were characterized by field-emission scanning electron microscopy (FE-SEM, Hitachi, S4800), transmission electron microscopy (TEM), high resolution transmission electron microscopy (HR-TEM) (TEM, JME-2100, Japan), and X-ray diffraction (XRD, MAC, M18XHF) using CuKα radiation (1.54 Å). Absorption spectra were obtained by a UV-Vis spectrophotometer (UV-2550, Shimadzu). Photoluminescence (PL) and Raman spectra were recorded by a micro-Raman spectroscopy system (Renishaw PLC in Via-Reflex). X-ray photoelectron spectroscopy (XPS, Thermo, ESCALAB 250) was employed to analyze the surface composition of the samples. The elemental distributions and concentrations were analyzed by Energynergy dispersive spectroscopy (EDS, Oxford Inca) accompanying the FE-SEM.

### Photoelectrochemical measurements

Photoelectrochemical measurements were carried out in a three-electrode configuration with the as-prepared sample as the working electrode, Pt foil as the counter electrode, and saturated Ag/AgCl as the reference electrode^41^. A 0.1 M Na_2_SO_3_ aqueous solution was used as the electrolyte. Photocurrent measurements were taken as a function of voltage by an electrochemistry workstation (CHI 660D, Shanghai Chenhua instrument). The working electrode was illuminated by a 300 W Xe lamp. An ultraviolet cutoff filter was inserted in between the light source and the quartz cell to exclude UV light with wavelength below 420 nm. Photoresponses of the different samples were determined by using a light on-off cycle of 60 s at a bias of 0 V versus the Ag/AgCl electrode.

## Results and Discussion

### XRD patterns

**Figure 1:**
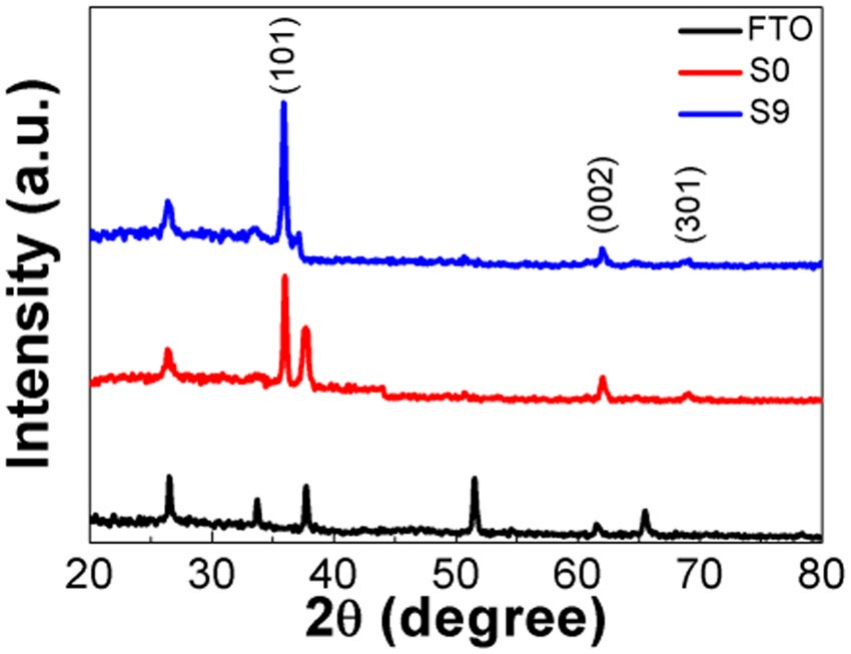
XRD patterns of S0, S9, and FTO substrate.

Figure 1 shows XRD patterns of S0 and S9. The XRD pattern of the substrate (FTO) was used as a reference. After the TiO_2_ products were formed on the FTO substrates, all FTO diffraction peaks became weaker. Both S0 and S9 samples have similar patterns, in that they display three peaks at 2θ = 36.02°, 62.56°, and 69.01°. These peaks can be attributed to the (101), (002), and (301) diffraction peaks of anatase TiO_2_, respectively, as they are in good agreement with the standard pattern of anatase TiO_2_ (PDF#65-1119). In addition, the diffraction peaks of Cu_x_O QDs are not observed in S9, implying the low content and small size of QDs. Therefore, we can conclude that the formation of Cu_x_O QDs does not influence the crystalline structure of the TiO_2_ electrodes.

### Morphological analysis

**Figure 2:**
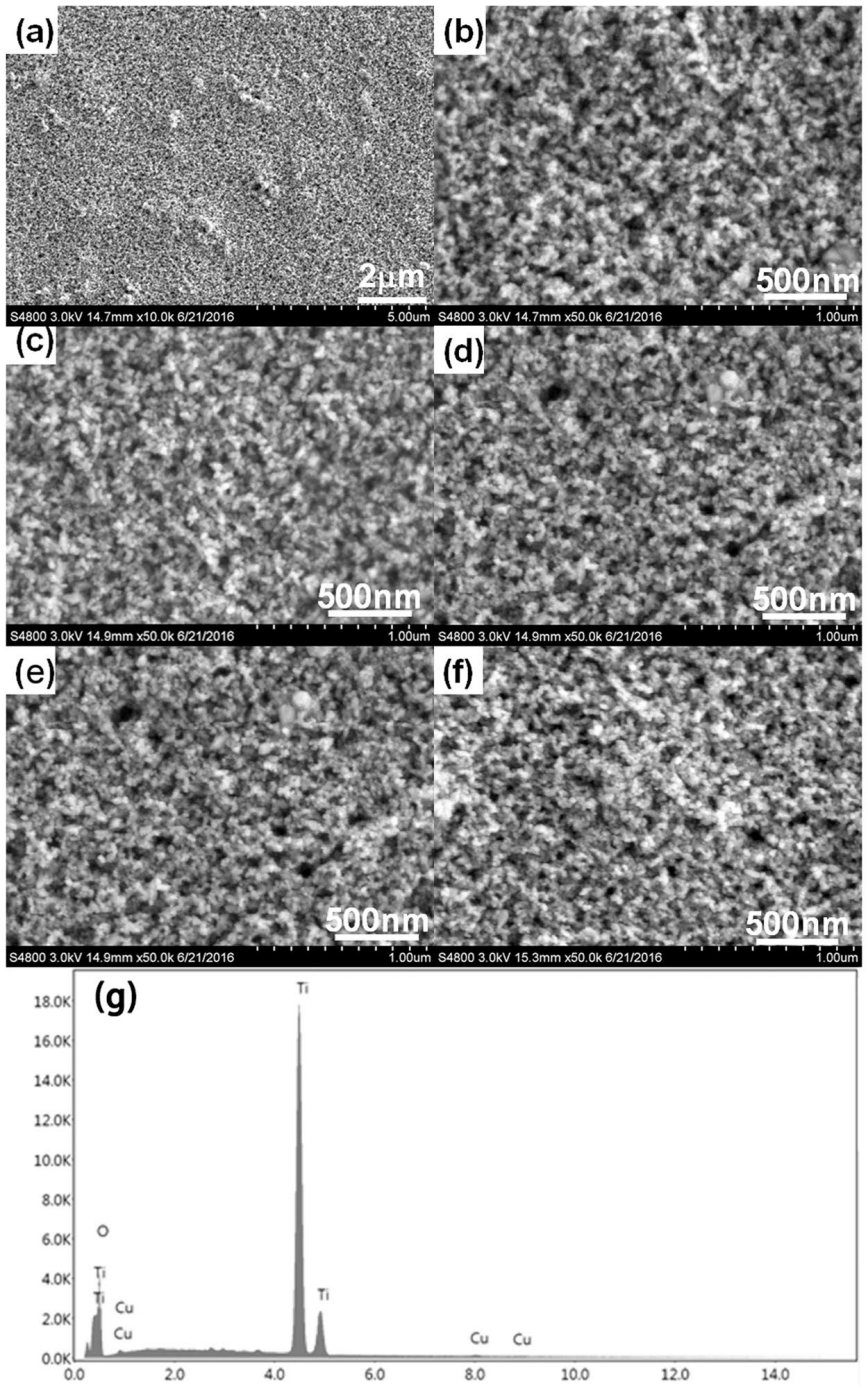
SEM images of S0 (**a**) close-up view (**b**) high resolution view (**c**) S3, (**d**) S5, (**e**) S7, (**f**) S9; and (**g**) EDS spectrum of S7.

Figure 2 presents SEM images of TiO_2_ films coated with CuxO QDs. The magnification and high magnification SEM images (Fig. 2a and b) show that the TiO_2_ film is porous and uniform. Figure 2c–f shows SEM images of the TiO_2_ after depositing 3, 5, 7, and 9 cycles of Cu_x_O QDs. No morphological changes were observed among the samples deposited with Cu_x_O QDs, due to the low content and small size of the Cu_x_O QDs; however, EDS data of S7 (Fig. 2(g)) reveal that the sample consists of Ti, O, and Cu elements, confirming the presence of Cu_x_O QDs.

**Figure 3:**
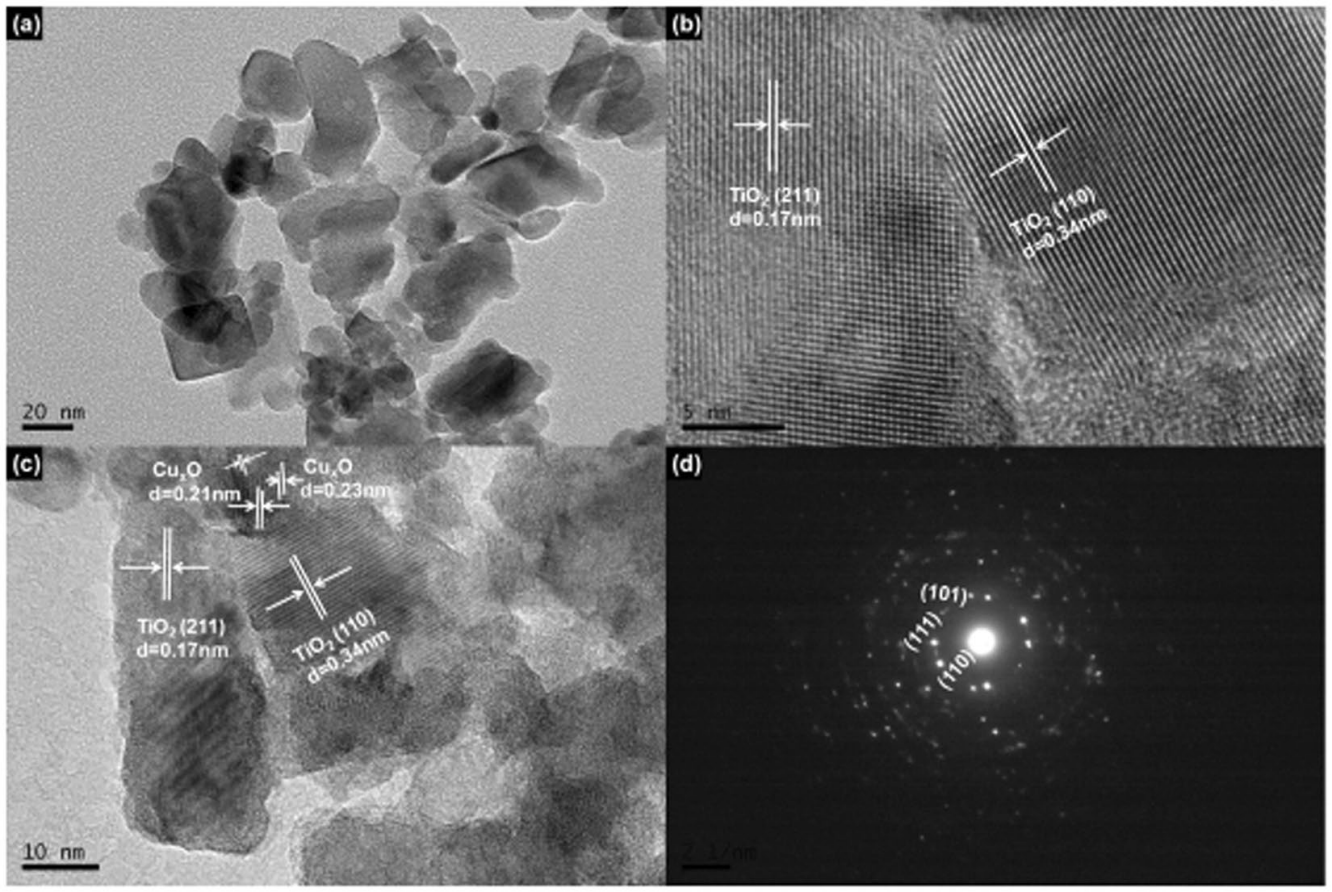
(**a**) TEM images of Cu_x_O/TiO_2_ (S7), (**b**) HRTEM of TiO_2_ (S0), (**c**) HRTEM of Cu_x_O/TiO_2_ (S7), and SAED pattern of the same Cu_x_O/TiO_2_ in part (**a**).

TEM and SAED characterization was employed to examine the crystal structure and growth direction of TiO_2_ as well as the particle size of the Cu_x_O QDs. Figure 3(a) shows that Cu_x_O QDs were in contact with the TiO_2_ film. The size of TiO_2_ nanoparticles and Cu_x_O QDs were approximately 34 nm and 10 nm, respectively. When the imaging was focused around the TiO_2_ and TiO_2_/Cu_x_O interface, various crystalline facets were clearly observed, as shown in Fig. 3b and c. The larger crystalline region in Fig. 3b was confirmed to be TiO_2_. The observed lattice plane spacings are 0.17 nm and 0.34 nm corresponding to the (211) and (110) planes of anatase TiO_2_. Above the anatase TiO_2_ crystallites, we also observed the interplanar spacings of 0.21 and 0.23 nm (Fig. 3c), which could be indexed to elementary Cu (111) (d = 0.21 nm), CuO (111) (d = 0.23 nm), or Cu_2_O (002) (d = 0.21 nm). In addition, the SAED pattern in Fig. 3d demonstrates that the TiO_2_ electrodes exhibit a single crystal structure.

### XPS analysis

**Figure 4:**
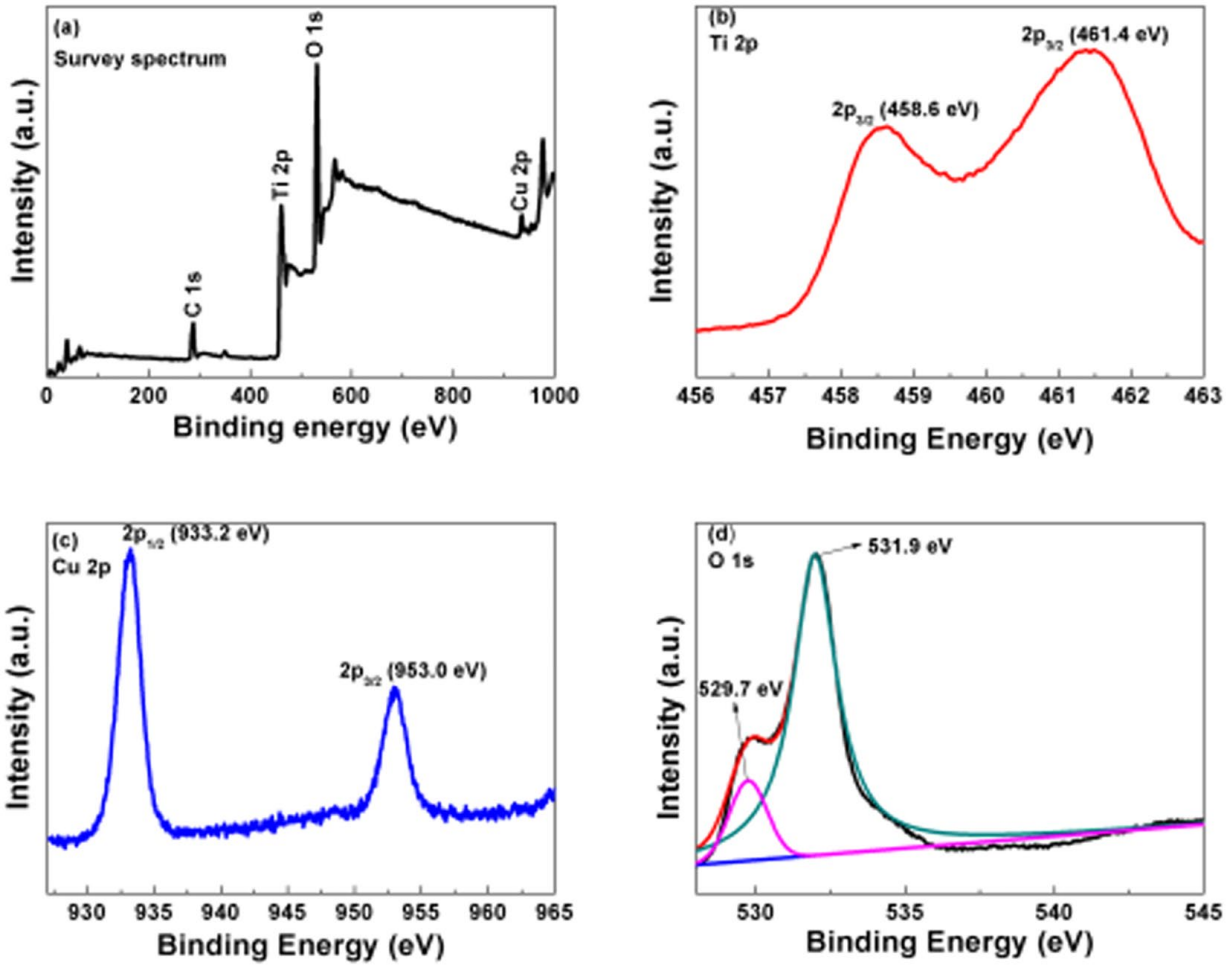
XPS spectra of S7: (**a**) Survey spectrum (**b**) Ti 2p, (**c**) Cu 2p, and (**d**) O 1s.

In order to clarify the elemental composition and valence state of Cu_x_O QDs, XPS characterizations was conducted. As shown in Fig. 4a, the general survey spectrum for Cu_x_O QDs modified TiO_2_ electrodes contains Cu, O, Ti, and C elements. The small amount of carbon could have resulted from adventitious hydrocarbons from the XPS instrument itself and can be taken as the standard signal for the correction of other peaks^42^. From the Ti 2p spectrum (Fig. 4b), two main peaks at bonding energies of 458.6 and 461.4 eV were assigned to Ti 2p_3/2_ and Ti 2p_1/2_, respectively^43, 44^. Figure 4c shows a representative Cu 2p core level XPS spectrum with two peaks at 933.2 and 953.0 eV for atmospheric conditions at room temperature, and the oxidation products include Cu_2_O and CuO^45, 46^. Furthermore, two fitted peaks (Fig. 4d) from the O 1 s spectrum are observed around 529.7 and 531.9 eV, which can be assigned to the lattice oxygen and surface hydroxyl oxygen of TiO_2_^47^, respectively.

### UV-Visible absorption spectra

**Figure 5:**
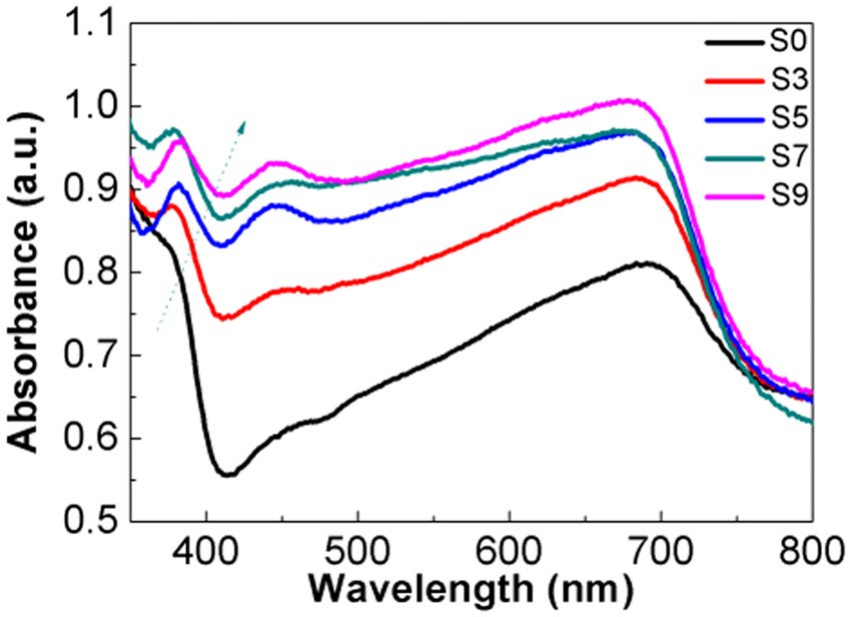
UV-Visible absorption spectra of Cu_x_O/TiO_2_ prepared with different SILAR cycles.

**Figure 6:**
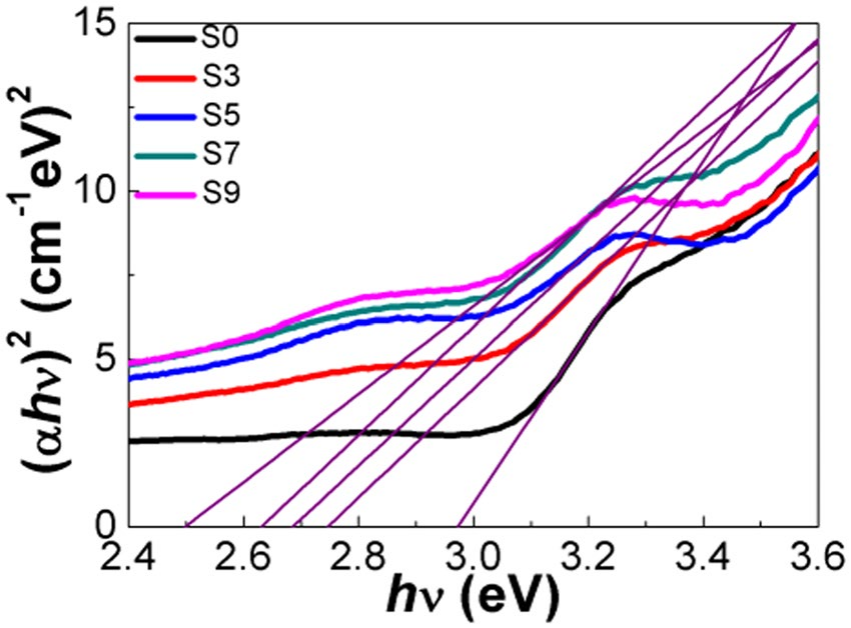
Plots of (*αhv*)^2^ versus energy (*hv*) for Cu_x_O/TiO_2_ prepared with different SILAR cycles.

Figure 5 shows the absorption spectra of TiO_2_ electrodes sensitized with different SILAR cycles of Cu_x_O QDs. The average absorbance can be calculated and the results are listed in Table 1. It can be seen that with an increase in the number of SILAR cycles, the absorbance increases at wavelengths 400 to 700 nm. This can be attributed to the SPR of Cu QDs and narrow band gap of CuO and Cu_2_O^48^. In addition, the red-shift of the absorption edge of Cu_x_O/TiO_2_ is due to the broadening of size distribution of Cu_x_O QDs^49^. Based on the UV-Visible absorption spectra, a plot of (*αhv*)^2^ versus energy (*hv*) is shown in Fig. 6, and the Eg values of different Cu_x_O samples are shown in Table 1. It can be seen that the absorption bands of Cu_x_O/TiO_2_ show large variation, which change from 2.90 to 2.50 eV. The band gap of Cu_x_O/TiO_2_ (S9) was 2.50 eV, which was smaller than that of TiO_2_ (2.90 eV). These results suggest that the formation of the Cu_x_O/TiO_2_ nanostructures decreased the recombination of photogenerated electrons and holes and improved the photoelectrochemical ability of the TiO_2_ electrodes.

**Table 1:** The average absorbance with 400–700 nm, band gap, and photocurrent values of TiO_2_/Cu_x_O electrodes.

Samples	Average absorbance (a.u.)	Band gap (eV)	Photocurrent values (dark) (μA/cm^2^)	Photocurrent values (light) (μA/cm^2^)
S0	0.68	2.98	37.60	168.88
S3	0.82	2.75	97.47	248.95
S5	0.89	2.69	123.79	390.98
S7	0.92	2.63	166.74	500.01
S9	0.94	2.50	145.30	424.15

### PL spectra

**Figure 7:**
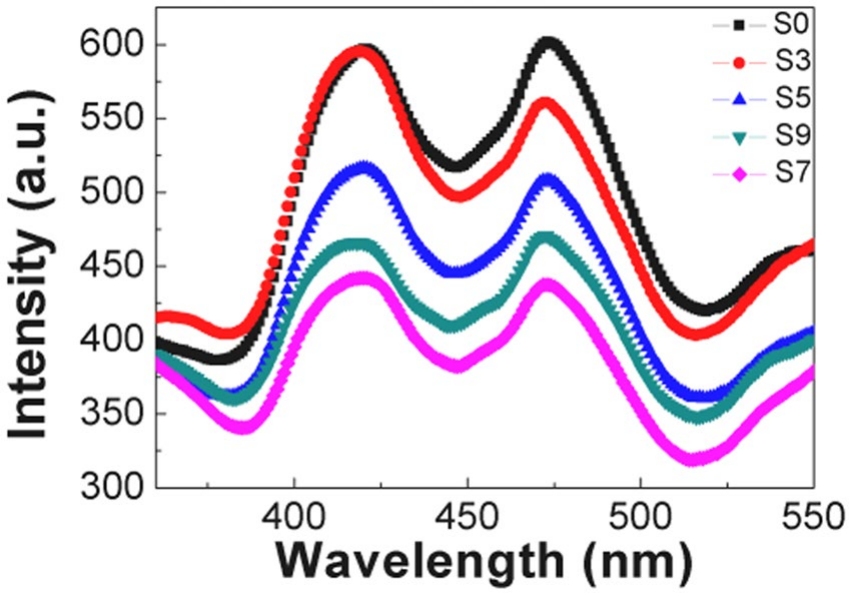
PL spectra of different samples.

To investigate charge transfer between photogenerated electctrons and hole pairs, photoluminescence (PL) emission spectroscopy was used to measure the recombination of free charge carriers. The emission peaks at 420 and 475 nm are assigned to exciton-casued PL resulting from band edge free excitons and defects of TiO_2_^50^. PL peak intensity correlates directly with the defect densities in materials. The higher PL intensity typically indicates a higher recombination rate of the photo-generated electrons and holes^51^. As shown in Fig. 7, the PL intensity of Cu_x_O/TiO_2_ reveals a significant decreases with increasing Cu_x_O QDs. This is due to a decrease of radiative recombination processes^52^. When the Cu_x_O QDs are deposited on TiO_2_ electrodes, TiO_2_ can easily bond with the Cu_x_O QDs to form the Cu_x_O/TiO_2_ composites. The photo-induced electrons can be trapped in the Cu 2p energy level below the conduction band in the Cu_x_O/TiO_2_ composites, which inhibit the recombination of electron-hole pairs. The intensity of the peaks at 420 and 475 nm are the lowest for S7, which exhibit high quantum efficiency and higher photoelectrochemical properties.

### Photoelectrochemical studies

**Figure 8:**
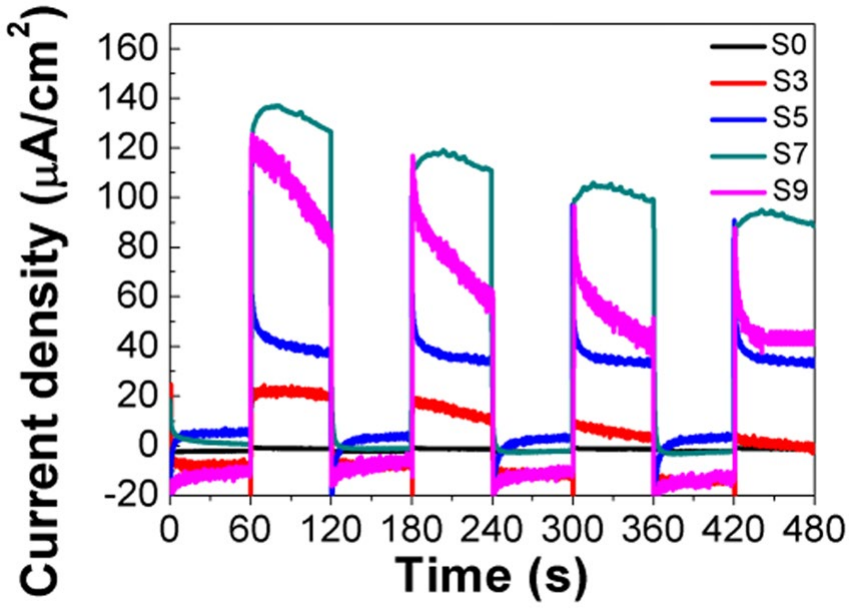
Transient photocurrents of Cu_x_O/TiO_2_ prepared with different SILAR cycles.

Figure 8 shows the time-dependent photocurrent curves of Cu_x_O/TiO_2_ under visible light illumination. It is generally known that transient photocurrent always reflect the transfer and separation of photoinduced charge carriers under intermittent illumination. As the light is turned on, the photocurrent values increase while the photocurrent values decrease rapidly as the light is turned off. This suggests that all samples have good reproducibility. In addition, photocurrents increase with more SILAR cycles, which indicate that the photocurrent of Cu_x_O/TiO_2_ have a significant enhancement compared to bare TiO_2_. Moreover, after 7 SILAR cycles, the sample shows the highest photocurrent value of ca. 138 μA/cm^2^, which is about 13 times higher than that for bare TiO_2_. Nevertheless, when the SILAR cycles increase to 9, the photocurrent value decreases to ca. 120 μA/cm^2^. The increase in photocurrent may be attributed to the stronger SPR effect of Cu_x_O QDs, which improves the light absorption of TiO_2_. With more than 9 SILAR cycles, the aggregation of Cu_x_O QDs lead to a large particle size of Cu_x_O QDs, which could block the surface active sites of TiO_2_ and act as potential barrier of charge transfer, resulting in a decrease of the photoelectrochemical properties^53–56^.

**Figure 9:**
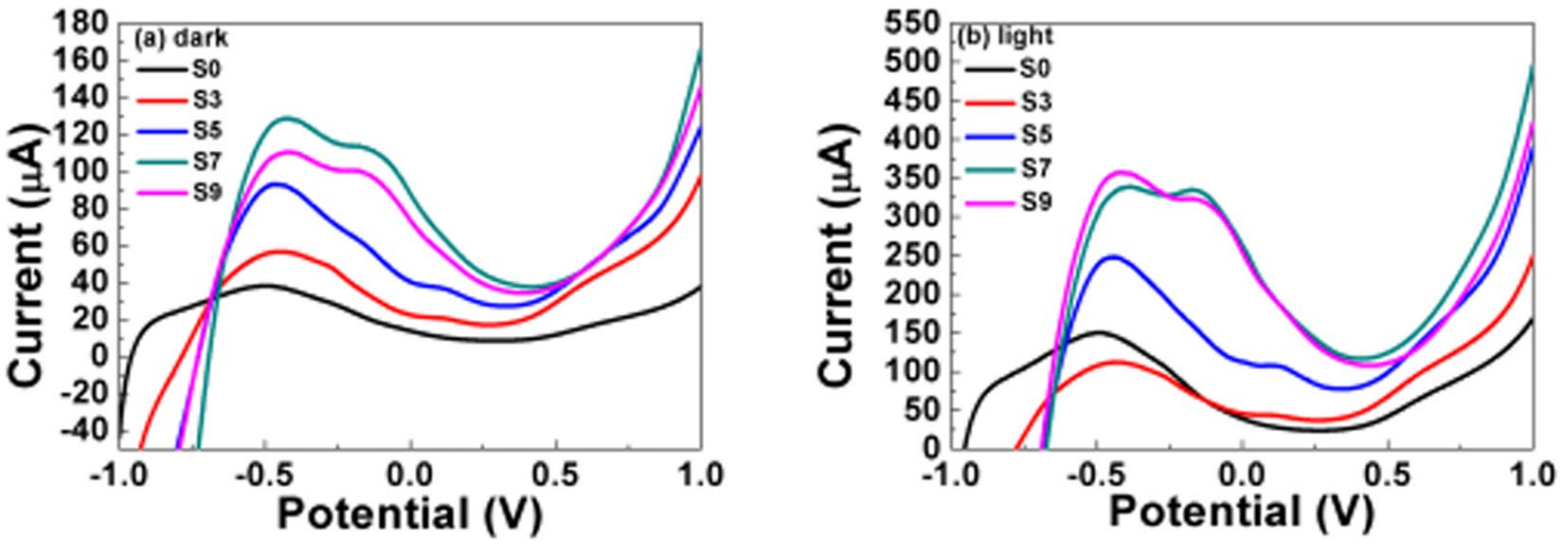
LSV spectra of Cu_x_O/TiO_2_ electrodes in dark and under visible light irradiation.

Figure 9 shows the LSV curves of the different samples in the dark and under light irradiation. Photocurrent values are shown in Table 1. The photocurrents of the Cu_x_O/TiO_2_ samples improved compared with that of bare TiO_2_, suggesting that the Cu_x_O/TiO_2_ exhibit a stronger ability to separate photo-generated electron-hole pairs. The Cu_x_O/TiO_2_ prepared with a different number of SILAR cycles (0, 3, 5, 7 and 9 times) exhibited photocurrent values of 168.88, 248.95, 390.98, 500.01, and 424.15 μA/cm^2^ at 1.0 V (vs Ag/AgCl) under visible light irradiation, respectively. Clearly, photocurrent densities of the Cu_x_O/TiO_2_ first increase then decrease with increasing the SILAR cycles. The S7 showed the strongest photocurrent value, and exhibits the best photoelectrochemical property, which is consistent with the PL results. When 9 SILAR cycles were used, the CuxO QDs aggregated to form a compact granular morphology, resulting in a lower surface area and reduced photocurrent^57^.

## Conclusions

In summary, Cu_x_O QDs are deposited on the nanoporous anatase TiO_2_ films by a screen-printing method, followed by successive ionic adsorption and reaction (SILAR). The microstructure, morphology, and loading amounts of the Cu_x_O QDs on the TiO_2_ films are controlled by changing the number of SILAR cycles. The Cu_x_O/TiO_2_ absorbs more light and exhibits enhanced photoelectrochemical properties compared to bare TiO_2_. Moreover, under visible light illumination, the TiO_2_ sensitized with 7 SILAR cycles of Cu_x_O QDs shows the best photoelectrochemical properties, where the photocurrent density is increased to 500.01 μA/cm^2^, 2.96 times higher than the bare TiO_2_ electrode with 168.88 μA/cm^2^. The superior photoelectrochemical properties of the Cu_x_O/TiO_2_ nanostructures could be ascribed to the large surface area of nanoporous TiO_2_ electrode and the SPR effect of Cu_x_O QDs. The electrode design of Cu_x_O/TiO_2_ will be beneficial for application of solar energy conversion and wastewater degradation.
